# 

**Published:** 1848

**Authors:** 


### NOTICE TO READERS AND CORRESPONDENTS.

We have received original articles from Drs. R. R. Stone, and J. T. Plummer, which shall appear in our next.

The following books have been received from Messrs. Lea & Blanchard, publishers of Philadelphia, which are for sale by Jos. KEEN, jr., Chicago:

A Dispensatory and Therapeutical Remembrancer, &c., by John Mayne, M.D., &c , Edinburg. Revised, &c., by R. Eaglesfield Griffith, M.D., &c. &c.

Elements of Natural Philosophy. By Goulding Bird, A.M., M.D., &c.

The Human Brain, &c. By Samuel Solly, F.R.S., &c.

On Disorders of the Cerebral Circulation, &c., with colored plates. By George Burrows, M.D.

Lallemand on Spermatorrhœa. Translated and edited by H. J. McDougal, M.R.C.S.E., &c.

On the Blood and Urine. By J. W. Griffith, M.D., &c., G. O. Reese, M.D., &c., and Alfred Marwick, M.D., &c.

On the Causes and Treatment of Abortion and Sterility, &c. By Jas. Whitehead, F. R. C. S., &c.

Practical Observations on Certain Diseases of the Chest, &c. By Peyton Blakiston, M.D., F.R.S., &c.

On Poison in relation to Medical Jurisprudence and Medicine. By Alfred S. Taylor, F.R.S., &c. Edited with notes by R. E. Griffith, M.D., &c.

The Practice of Medicine, &c. By R. Dunglison, M.D., Prof., &c., 3d edition, 2 vols.

Lectures on the Physical Phenomena of Human Beings. By Carlo Mattucci, Prof., &c., Pisa. Translated under the supervision of J. Pereira, M.D., &c.

Also, from the author, Theory of Human Existence. By T. S. Wright, M.D., Cincinnati.

Catalogues of Medical Colleges, Introductory Lectures, Dr. Cartwright's Address, our usual Exchanges, &c., which our room will not allow us to notice in this number.

### RECEIPTS FOR THE MEDICAL JOURNAL FOR MAY AND JUNE, 1848.

### ILLINOIS.

| | |
|---|---|
| Dr H. H. Sexton, Galesburg, | $4 00 |
| Dr A. Mosher, Pleasant Hill, | 5 00 |
| Dr John Riches, Elizabeth, | 3 00 |
| Dr D. Ward, Ottawa, | 4 00 |
| Dr S. Meacham, Monroe, | 2 00 |
| Dr W. W. Welch, Inlet, | 2 00 |
| Dr C. C. Bradly, Byron, | 2 00 |
| Dr E. G. Hough, Downer's Grove, | 2 00 |
| Dr A. W. Heisse, Addison, | 1 00 |
| Dr L. F. Avery, Babcock's Grove. | 1 00 |
| Dr Wm. Adams, McMillen's Grove, | 1 00 |
| Dr D. Burton, Algonquin, | 1 00 |
| Dr — Hale, Dundee, | 2 00 |
| Dr Scofield, Yorkville, | 2 00 |
| Dr A. W. King, Woodstock, | 1 00 |
| Dr H. Cutler, Barbour's Grove, | 1 00 |
| Dr A. Smith, Lyndon, | 2 00 |
| Dr E. S. L. Richardson, Bristol, | 4 00 |
| Dr — Delany, Solon Mills, | 2 00 |
| Dr E. S. Morey, Rock Island, | 2 00 |
| Dr L. A. Hanneford, Trivoli, | 2 00 |
| Dr M. Wright, Amesville, | 2 00 |
| Dr R. F. Adams, Lee Centre, | 2 00 |
| Dr E. M. Garfield, Dresden, | 2 00 |
| Dr C. C. Barnes, Naperville, | 1 00 |
| Dr M. Bacon, Otsego, | 2 00 |
| Dr J. Bennett, Iraquois, | 2 00 |
| Dr J. Q. Newton, Warrenville, | 2 00 |
| Dr B. R. Hart, Alton, | 2 00 |

### INDIANA.

| | |
|---|---|
| Dr O. H. Martin, Andersonville, | 4 00 |
| Dr N. Sherman, Plymouth, | 4 00 |
| Dr C. Brackett, Rochester, | 2 00 |
| Dr A. W. Dewey, Benton, | 2 00 |
| Dr E. H. Drake, Brookville, | 2 00 |
| Dr A. Wolverton, America, | 1 00 |
| Dr R. H. Tarleton, Martinsville, | 2 00 |
| Dr John Sims, Aboit, | 1 00 |
| Dr Wm. Townsend, Royal Centre, | 2 00 |
| Dr J. H. Constant, Mexico, | 2 00 |
| Dr T. D. McCrea, Winnemac, | 2 00 |
| Dr G. P. Durr, Kewana, | 2 00 |
| Dr J. Pennington, Cambridge City, | 2 00 |
| Dr — West, Hagerstown, | 2 00 |
| Dr -- Buchannan, Hagerstown, | 2 00 |
| Dr J. Bell, Milton, | 4 00 |
| Dr T. T. Butler, Noblesville, | 2 00 |
| Dr M. G. Walker, Pendleton, | 2 00 |
| Dr — Haines, Westfield, | 2 00 |
| Dr — Davis, Westfield, | 4 00 |
| Dr A. G. Ruddle, Allisonville. | 2 00 |
| Dr I. Maranda, Allisonville, | 2 00 |
| Drs Boor & Guissinger, Middletown. | 2 00 |
| Dr J. C. Helm, Granville, | 2 00 |
| Dr W. C. Willard, Muncietown, | 2 00 |
| Dr S. P. Anthony, Muncietown, | 2 00 |
| Dr H. Carver, Economy, | 2 00 |
| Drs Maulsby & Robins, Economy, | 2 00 |
| Dr D. S. McGaughey, Morristown, | 2 00 |
| Dr A. J. Mullen, Napoleon, | 4 00 |
| Dr J. H. Wiley, St. Omar, | 2 00 |
| Dr W. W. Taylor, Vienna, | 1 00 |
| Dr E. Clifford, Falmouth, | 2 00 |
| Dr G. Watt, Bentonville, | 2 00 |
| Dr U. B. Tingley, Harrisburg, | 2 00 |
| Dr P. Trimbly, Brownsville, | 2 00 |
| Dr W. J. Walker, Dunlapsville, | 2 00 |
| Dr C. C. Pease, Sunman's P. O., | 2 00 |
| Dr R. C. Bond, Sunman's, P. O, | 2 00 |
| Dr D. Tevis, Moscow, | 2 00 |
| Dr W. C. Robinson, Moscow, | 2 00 |
| Dr M. Robins, Shelbyville, | 2 00 |
| Drs Webb & Day, Shelbyville, | 1 00 |
| Dr M. W. Thomas, Franklin, | 2 00 |
| Dr J. McCorcle, Franklin, | 2 00 |
| Dr S. M. Linton, Columbus, | 2 00 |
| Dr S. Jackson, Columbus, | 2 00 |
| Dr H. D. Henderson, Salem, | 2 00 |
| Dr E. Newland, Salem, | 2 00 |
| Dr J. H. Shanks, Salem, | 2 00 |
| Drs Read & Beck, Salem, | 2 00 |
| Dr B. Newland, Bedford, | 1 00 |
| Dr J. Stilson, Bedford, | 1 00 |
| Dr R. C. Hamills, Bloomington, | 2 00 |
| Dr. C. F. Butler, Far West, | 1 00 |
| Dr. W. C. Thompson, Indianapolis, | 2 00 |
| Dr. E. Horner, Indianapolis, | 2 00 |
| Dr. Lot Edwards, Greenfield, | 2 00 |
| Dr. G. M. Huggans, Darlington, | 2 00 |
| Dr. J. Pritchett, Centreville, | 2 00 |
| Dr. M. D. Frazier, Yorktown, | 2 00 |
| Dr. S. Hicks, Napoleon, | 2 00 |
| Dr. S. T. Cowgill, Greencastle, | 2 00 |
| Dr. D. L. Bradley, Laporte, | 4 00 |
| Dr. R. Lee, Palestine, | 2 00 |
| Dr. T. Adamson, Williamsburg, | 2 00 |
| Dr. — Smith, Richmond, | 2 00 |

### WISCONSIN.

| | |
|---|---|
| Dr. Jeremiah Look, Waupun, | 2 00 |
| Dr. C. B. Chapman, Madison, | 2 00 |
| Dr. C. C. Warren, Walworth, | 1 00 |
| Dr. D. C. Houndy, Sharron, | 1 00 |
| Drs. Johnson & Terwilliger, Palmyra, | 1 00 |
| Dr. R. Patee, Palmyra. | 2 00 |
| Dr. A. Squires, Rochester, | 1 00 |
| Dr. W. Hankardt, Rochester, | 1 00 |
| Dr. D. Robinson, Rochester, | 1 00 |
| Dr. B. Salisbury, Eaglevillage. | 1 00 |
| Dr. R. Dunlap, Waukesha, | 2 00 |
| Dr. E. B. West, Waukesha, | 2 00 |
| Dr. J. M. Todd, Waukesha, | 2 00 |
| Dr. Wm. Gorham, Milwaukie, | 2 00 |
| Drs. Woodward & McHenry, Southport, | 2 00 |
| Dr. P. P. Green, Southport, | 2 00 |
| Dr. S. Blood, Rochester, | 2 00 |
| Dr. G. Wright, Waukesha, | 3 00 |
| Dr. M. K. Fowler, Milwaukie, | 2 00 |
| Dr. E. S. Marsh, | 4 00 |
| Dr. — Wadsworth, Racine, | 2 00 |
| Drs. A. & E. N. Clark, Beloit, | 2 00 |
| Dr. J. C. Mills, Clinton, | 1 00 |
| Dr. W. W. Blackman, Johnstown, | 1 00 |
| Dr. E. Rider, Milton, | 2 00 |
| Dr. B. F. Collins, Fulton, | 2 00 |
| Dr. J. B. Waterbury, Verona, | 1 00 |
| Dr. S. W. Abbott, Madison, | 2 00 |
| Dr. S. F. Gibbs, College Grove, | 1 00 |
| Dr. C. Pratt, Sun Prairie, | 50 |
| Dr. Ira R. Rood, Waterloo, | 2 00 |
| Dr. J. L. Williams, Columbus, | 1 00 |
| Dr. J. Prentice, Fox Lake, | 1 00 |
| Dr. J. W. Kimball, Beaver Dam, | 2 00 |
| Dr. B. Noyes, Beaver Dam | 50 |
| Dr. A. Atwood, Oak Grove, | 1 00 |
| Dr. J. R. Goodno, Watertown, | 2 00 |
| Drs. Lyman & Johnston, Jefferson, | 2 00 |
| Dr. J. Winslow, Fort Atkinson, | 4 00 |
| Dr J. W. Brown, Waupun, | 3 00 |
| Dr W. H. Walker, Fon du Lac | 2 00 |

### IOWA.

| | |
|---|---|
| Dr. E. Kirkup, Springfield, | 2 00 |

### MICHIGAN.

| | |
|---|---|
| Dr. — Romine, Colon, | 2 00 |
| Dr. J. S. Skinner, Litchfield, | 2 00 |

### NEW YORK.

| | |
|---|---|
| Dr. T. R. Beck, Albany, | 4 00 |
| Albany Med. College, Albany, | 2 00 |

